# The Cure for Hydrophobia

**Published:** 1882-10

**Authors:** 


THE CURE FOR HYDROPHOBIA.—Some months ago, a French physician excited much attention by reporting a case of hydrophobia in man, which was apparently cured by pilocarpin.

More recently, M. Decroix, an eminent veterinarian, has reported a series of cases of rabies occurring in men and dogs. He asserts, very positively, that the disease is sometimes spontaneously curable, both in man and other animals. He thinks that the administration of medicines does more harm than good.

Filing the teeth of dogs is recommended as a better safeguard than muzzling.

It is apparent that the pathology of rabies is still far from being settled.

